# Association of HbA_1c_ and an updated glucose management indicator (uGMI) with incident diabetic retinopathy in adults with type 1 diabetes: a longitudinal study

**DOI:** 10.1007/s00125-025-06599-w

**Published:** 2025-11-11

**Authors:** Viral N. Shah, Yongjin Xu, Yaghoub Dabiri, Hemanth P. Mohanadas, Alan Cheng, Timothy C. Dunn

**Affiliations:** 1 Division of Endocrinology & Metabolism, Indiana University School of Medicine, Indianapolis, IN USA; 2 Center for Diabetes and Metabolic Diseases, Indiana University School of Medicine, Indianapolis, IN USA; 3 Abbott Diabetes Care, Alameda, CA USA

**Keywords:** CGM, Diabetic retinopathy, HbA_1c_, Type 1 diabetes, Updated GMI

## Abstract

**Aims/hypothesis:**

This study aimed to compare the predictive performance of HbA_1c_ and a continuous glucose monitoring (CGM)-based updated glucose management indicator (uGMI) in assessing incident diabetic retinopathy risk.

**Methods:**

We used the data from a previously published longitudinal case–control study that collected CGM data for up to 7 years prior to diagnosis of incident diabetic retinopathy or no retinopathy (control participants) among adults with type 1 diabetes. Mutual information scores (MIS), receiver operating characteristics (ROC) curves and machine learning models were used to assess the associations of diabetic retinopathy with HbA_1c_, uGMI and CGM-derived metrics.

**Results:**

The uGMI demonstrated a stronger association with incident diabetic retinopathy (MIS 0.148) compared with HbA_1c_ (MIS 0.078). ROC analysis showed that uGMI had a modestly higher AUC (AUC 0.733) than HbA_1c_ (AUC 0.704). Decision tree models incorporating both HbA_1c_ and uGMI did not improve clinically significant diabetic retinopathy risk prediction. Machine learning models confirmed the better predictive value of uGMI, especially for HbA_1c_ values between 54 mmol/mol (7.1% NGSP) and 58 mmol/mol (7.5% NGSP), where diabetic retinopathy risk escalated significantly.

**Conclusions/interpretation:**

The uGMI is a slightly stronger predictor of diabetic retinopathy risk compared with HbA_1c_. HbA_1c_ and uGMI do not appear to be complementary for diabetic retinopathy risk prediction.

**Graphical Abstract:**

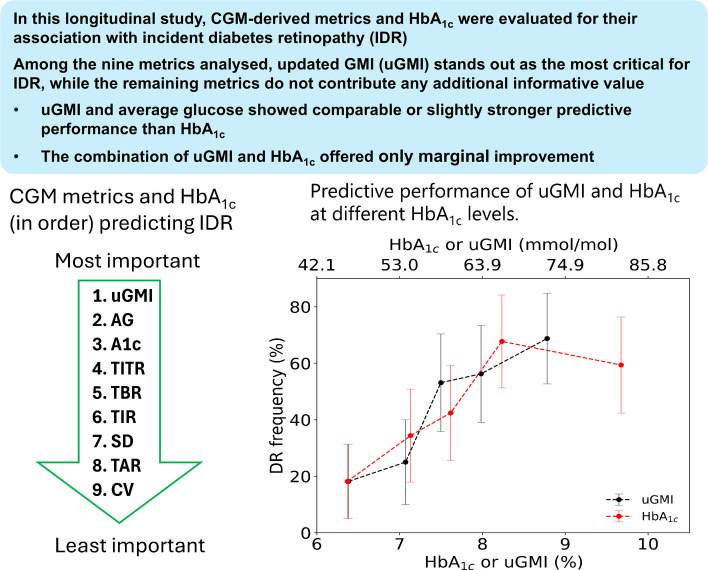

**Supplementary Information:**

The online version contains peer-reviewed but unedited supplementary material available at 10.1007/s00125-025-06599-w.


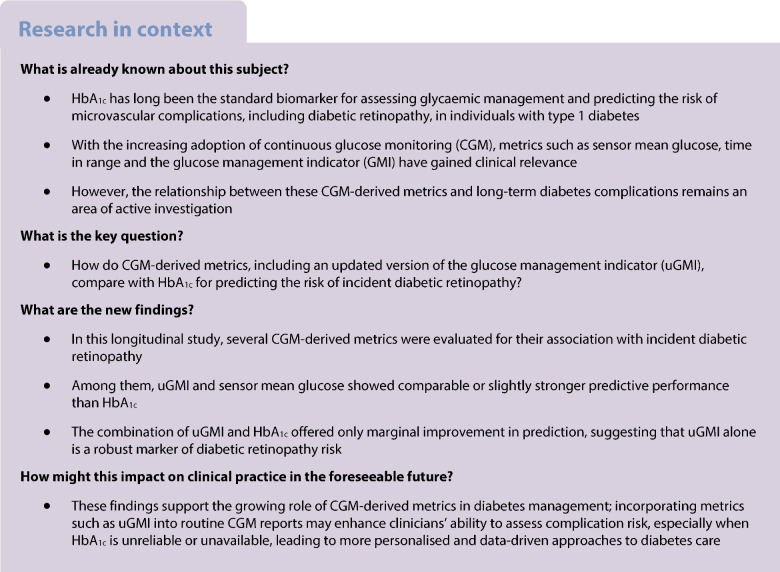


## Introduction

The DCCT provided compelling evidence for intensive glycaemic management to reduce microvascular complications in people with type 1 diabetes [1, 2]. Intensive insulin therapy, compared with conventional treatment, reduced the relative risk of diabetic retinopathy onset by 76% in the primary prevention cohort, and slowed its progression by 54% in the secondary intervention cohort [3, 4].

Since the DCCT trial, HbA_1c_ has become established as the gold standard marker for glycaemic management and microvascular complication risk in type 1 diabetes [5, 6]. HbA_1c_ provides a weighted average measure of blood glucose levels over the preceding 2–3 months [7]. Its clinical utility is well-established, offering a standardised, reproducible and validated predictor of diabetes-related complications [8]. However, HbA_1c_ alone does not provide detailed information on glycaemic profile and variations, and is confounded by factors such as erythrocyte turnover, haemoglobinopathies and individual biological variability, which may obscure a patient’s true glycaemic status [7].

With continuous glucose monitoring (CGM) becoming the standard of care in managing type 1 diabetes [9], CGM metrics and its goals have been included in the ADA Standards of Care 2025 [10]. Among these metrics, sensor mean glucose (AG), time in range (TIR: 3.9–10 mmol/l) and the glucose management indicator (GMI), an estimation of HbA_1c_ based on linear regression of mean glucose, have gained prominence [11, 12]. Studies have reported limitations of GMI, as it significantly deviates from HbA_1c_, especially at lower mean glucose [13, 14]. These studies have called for the GMI formula to be updated to provide a better representation of HbA_1c_ [13, 14].

The relationship between CGM metrics and diabetes complications remains an area of growing interest. By systematically comparing CGM-derived metrics with the established HbA_1c_ standard, this study seeks to address a critical gap in the literature. Glycaemic variability and glucose excursions, captured by coefficient of variation (CV) [15–17] and TIR [18], have been shown to contribute to retinal vascular damage. We assess the performance of CGM metrics, including an updated GMI (uGMI, details provided in Methods and electronic supplementary material [ESM] Methods), and explore the implications of integrating uGMI into clinical practice to reflect true glycaemic status and provide a complication risk estimate as for HbA_1c_.

## Methods

### Study design and participants

We used the data from a previously published longitudinal, case–control study [18]. The dataset included adults (age 18 years and above) with type 1 diabetes with who had a retinal examination documented between June 2018 and March 2022. Incident diabetic retinopathy was defined as diabetic retinopathy occurring during the study inclusion period in a patient in whom the two consecutive previous retinal examinations did not show retinopathy. Adults without diabetic retinopathy (control participants) were defined as those with absence of diabetic retinopathy during the study inclusion period, with two previous examinations without retinopathy. Details for defining diabetic retinopathy and collection of data have been published previously [18]. Pregnant individuals and those with incomplete CGM data were excluded to ensure dataset consistency. HbA_1c_ readings and other medical information were extracted from electronic medical records as detailed previously [18]. This was a retrospective study approved by University of Colorado institutional review board under the exempt category, and hence participant consent was not required for this research.

### CGM data collection and variables

For each participant, raw CGM data for up to 90 days and HbA_1c_ were collected at each clinic visit performed after 1 January 2013 and before the date of diagnosis of diabetic retinopathy (between June 2018 to March 2022). AG, uGMI, standard deviation of the glucose readings (SD), CV, TIR (3.9–10 mmol/l), time in tight range (TITR: 3.9–7.8 mmol/l), time above range (TAR: >10 mmol/l) and time below range (TBR: <3.9 mmol/l) were calculated from CGM data. Mean HbA_1c_ was calculated by averaging all available measurements for each participant. In this study, we updated the GMI calculation as: uGMI (mmol/mol) = 1/(0.07808/AG [mmol/l] + 0.003889) − 23.497, or in National Glycohemoglobin Standardization Program (NGSP) units: uGMI (%) = 1/(15.36/AG [mg/dl] + 0.0425). This formula is derived from our previously published study [19], where the ‘equilibrium HbA_1c_’, the expected HbA_1c_ under a steady-state glucose level, with reference kinetic constants of *k*_gly_=3.4 × 10^–7^ l mmol^–1^ day^–1^, *k*_age_=0.0095 day^–1^ and *K*_M_=8514 mmol/l, is now renamed as uGMI.

### Statistical analyses

#### Mutual information analysis

The mutual information score (MIS) [20] was used to assess the degree of association between selected variables and incident diabetic retinopathy. The MIS quantifies by how much uncertainty in one variable is reduced by knowing another. In this study, MIS for incident diabetic retinopathy were calculated for nine variables: uGMI, AG, HbA_1c_, TITR, TIR, TBR, TAR, SD and CV. The analysis was performed using the mutual_info_score module from the Python/scikit-learn library [21], providing a ranking of variables based on their predictive value. To identify a subset of features that are both highly relevant to the outcome and minimally redundant with one another, we applied the minimum redundancy maximum relevance (mRMR) algorithm [22]. In this approach, relevance is quantified using mutual information between each feature and the target variable, while redundancy is assessed based on mutual information among the features themselves. A positive mRMR score indicates that a feature provides more distinctive outcome-relevant information than it shares with other features, making it a valuable contributor to the model. Conversely, a negative score suggests that the feature is more redundant than informative, probably overlapping with information that has already been captured by features with positive scores. As the mRMR method is a univariate method for relevance and redundancy, it may underestimate the value of synergistic features that are only predictive when used together. For this reason, we also applied a recursive feature elimination method with a random forest model to confirm complementarity between features.

#### Cumulative rates and receiver operating characteristics (ROC) curves

The cumulative rate plot was employed to assess the effectiveness of various CGM metrics in classifying individuals with diabetic retinopathy (i.e. no diabetic retinopathy vs diabetic retinopathy). In this plot, the *x *axis represents the threshold values of the chosen metric (e.g. AG), while the *y* axis represents the cumulative rate of diabetic retinopathy. For each threshold, three curves were analysed: (1) the accuracy curve, which indicates the overall proportion of correct classifications (including both no diabetic retinopathy and diabetic retinopathy); (2) the true positive rate curve, which reflects the proportion of actual diabetic retinopathy diagnoses correctly identified at a given threshold (sensitivity); and (3) the false positive rate curve, which indicates the proportion of participants with no diabetic retinopathy who were incorrectly classified as having diabetic retinopathy. These curves collectively provide a detailed visualisation of the classification performance at various thresholds. The ROC curve further complements this analysis by examining the discriminative power of metrics such as AG and HbA_1c_ in distinguishing diabetic retinopathy from no diabetic retinopathy. The ROC curve plots the true positive rate on the *y *axis against the false positive rate on the *x *axis across a range of threshold values. A higher AUC value indicates a greater ability of the metric to differentiate accurately between diabetic retinopathy and no diabetic retinopathy within a threshold.

#### Decision tree analysis

Decision tree models were used to evaluate the association of HbA_1c_ with diabetic retinopathy independently and complementary to uGMI, which was the top CGM parameter identified in the previous step. Two decision tree models with one splitting node were constructed, with HbA_1c_ or uGMI, to compare the accuracy of using clinical thresholds. An additional two-layer decision tree was built with both variables, again using clinical thresholds, to evaluate the complementarity of using HbA_1c_ and uGMI.

#### Random forest and logistic regression

Random forest and logistic regression models [23–26] were used to investigate the individual and combined contributions of HbA_1c_ and uGMI to the diabetic retinopathy outcome. In this analysis, the independent variables are the mean HbA_1c_ and AG recorded prior to diagnosis for each individual. Specifically, we calculated the mean of all available HbA_1c_ and CGM readings, excluding any measurements taken after the diagnosis in the diabetic retinopathy group. The random forest models leveraged ensemble techniques to capture non-linear, complex relationships and interactions, while the logistic regression models focused on assessing linear dependencies and correlations. Both random forest and logistic regression were implemented using Python’s scikit-learn library [21], with cross-validation techniques used to optimise model parameters and validate performance. This approach facilitates a comprehensive understanding of the predictive power of uGMI alone, HbA_1c_ alone and their combined effect in classifying diabetic retinopathy, while highlighting potential interdependencies between the metrics.

All data processing and analysis were conducted using Python (version 3.8, downloaded from www.anaconda.com), using libraries including scikit-learn [21] for machine learning models and mutual information analysis.

## Results

### Baseline characteristics of participants

Over a median follow-up period of 2.8 years, 71 adults with type 1 diabetes developed diabetic retinopathy during the study period and 92 adults with type 1 diabetes remained free from diabetic retinopathy. Two individuals in the retinopathy-free group were excluded because of gaps in CGM data near the time of HbA_1c_ measurements. The baseline characteristics have been reported previously [16], and are provided in ESM Table 1. At baseline, the HbA_1c_ was 67 ± 13 mmol/mol or 8.3 ± 1.2% NGSP (mean ± SD) in the diabetic retinopathy group and 57 ± 12 mmol/mol or 7.4 ± 1.1% NGSP in the group without diabetic retinopathy. The corresponding AG were 10.3 ± 1.7 mmol/l and 9.1 ± 1.5 mmol/l, respectively. Among the participants, 60% used a Dexcom CGM (manufactured by Dexcom, San Diego, CA, USA), 19% used a Medtronic device (manufactured by Medtronic, Minneapolis, MN, USA), and 21% used multiple CGM technologies. One participant used a Libre sensor (manufactured by Abbott Diabetes Care, Alameda, CA, USA).

### Mutual information score

**Fig. 1:**
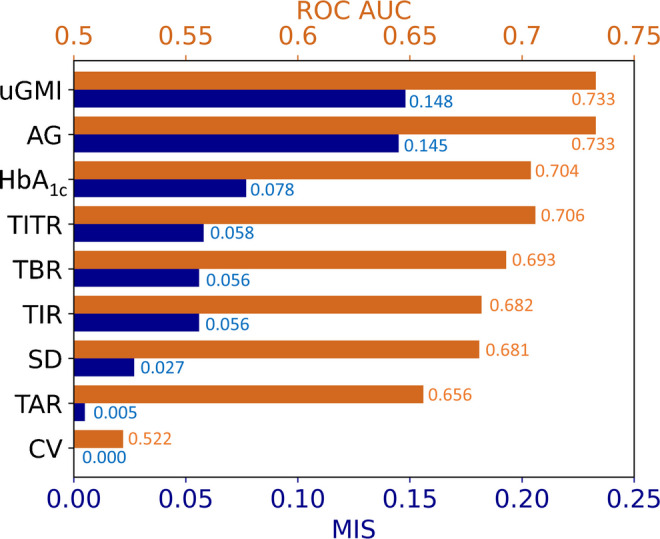
MIS (purple bars) and ROC AUC values (orange bars) for predictive metrics of diabetic retinopathy

The uGMI had the highest MIS (0.148), closely followed by AG (0.145), underscoring their strong association with diabetic retinopathy (Fig. 1). HbA_1c_ had a lower MIS of 0.078, suggesting it conveys less risk information than AG and uGMI. Among the CGM-derived metrics, TITR had the next highest MIS (0.058), followed by TBR (0.056) and TIR (0.056). The SD (MIS 0.027) contributed relatively less explanatory power, while the CV showed no association (MIS 0). The mRMR analysis identified uGMI as the feature with the highest relevance to diabetic retinopathy (ESM Fig. 1). In contrast, AG, SD, CV, TAR, TBR, TIR, TITR and HbA_1c_ all exhibited negative mRMR scores in the presence of uGMI, indicating that they contributed more redundancy than additional predictive value. To further assess feature complementarity, we applied recursive feature elimination using a random forest classifier. The results indicated that AG, SD, CV, TAR, TBR, TIR and TITR did not improve model performance when combined with uGMI, implying a lack of complementary predictive information. In contrast, HbA_1c_ demonstrated slight complementarity, as its inclusion alongside uGMI led to a slight improvement in model accuracy (ESM Fig. 2).

### ROC analysis

AG and uGMI achieved the highest AUC values of 0.733, followed by HbA_1c_ (0.704), indicating good performance in distinguishing between individuals with and without diabetic retinopathy (Fig. 1). While TITR had an AUC of 0.706, similar to that of HbA_1c_, TBR and TIR had lower AUC values (0.693 and 0.682, respectively).

### Relationship between CGM metrics and diabetic retinopathy frequency across percentile groups

The diabetic retinopathy risk was evaluated by grouping participants into five equal-sized bins based on their average HbA_1c_ or uGMI values. The average HbA_1c_ or uGMI and the corresponding percentage of patients with diabetic retinopathy were calculated and compared between the 1st and 4th quintiles and between the 2nd and 3rd quintiles in Table 1.

**Table 1:** Incident diabetic retinopathy across quintiles of uGMI and HbA_1c_

	uGMI		HbA_1c_	
	Mean (mmol/l)	DR risk (%)	Mean (mmol/l)	DR risk (%)
1st quintile	46	18	46	18
4th quintile	64	56	66	68
DR rate increase per 1% uGMI or HbA_1c_ increase (%)	24		28	
*p* value (between uGMI and HbA_1c_ rate increase)	0.15			
2nd quintile	54	25	54	34
3rd quintile	58	53	60	42
*p* value (between 2nd and 3rd quintile risks)	0.04*		0.68	

DR, diabetic retinopathy

^*^Statistically significant at *p*<0.05

**Fig. 2:**
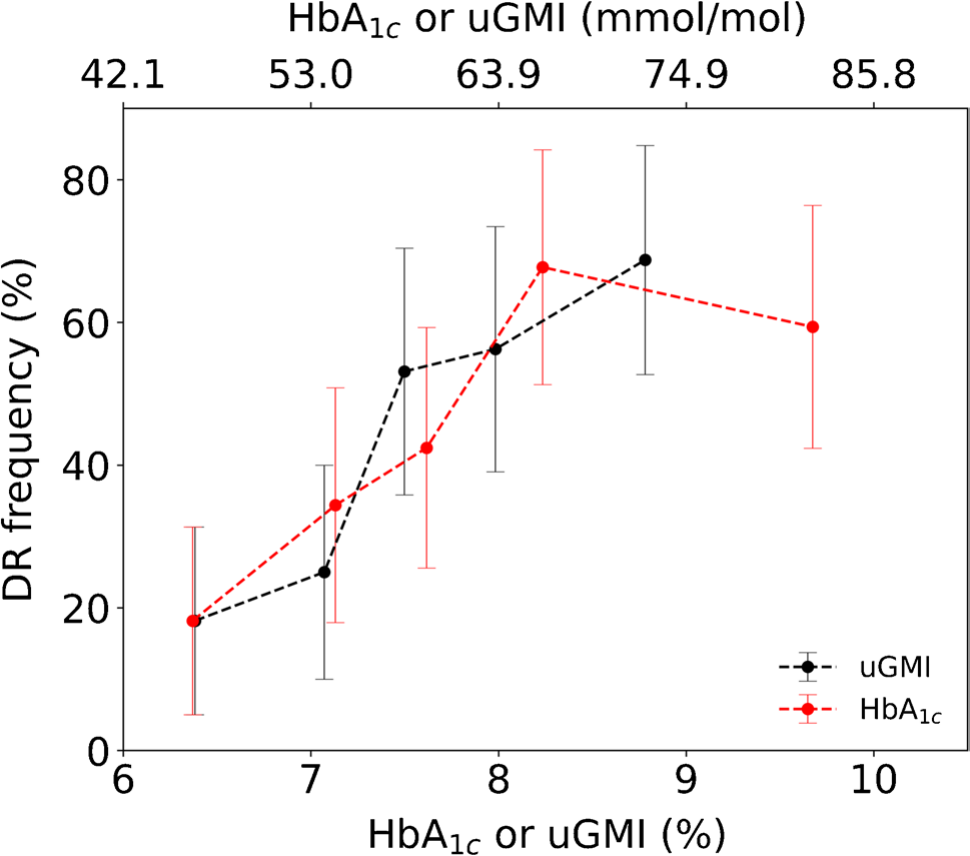
Comparison of diabetic retinopathy (DR) frequency across quintiles of uGMI and HbA_1c_. For every quintile, all pairwise *p* values for percentage diabetic retinopathy frequency between HbA_1c_ and uGMI are greater than 0.5

Between the first and fourth quintiles (uGMI 46–64 mmol/mol [6.4–8.0% NGSP]; HbA_1c_ 46–66 mmol/mol [6.4–8.2% NGSP]), each 11 mmol/mol (or 1% NGSP) increase in uGMI and HbA_1c_ was associated with a 24% and 28% increase in diabetic retinopathy risk, respectively (*p*=0.15). Notably, between the second and third quintiles, the diabetic retinopathy risk rose sharply from 25% to 53% (*p*=0.04) as uGMI increased from 54 to 58 mmol/mol (7.1–7.5% NGSP). In contrast, the diabetic retinopathy risk increased only modestly from 34% to 42% (*p*=0.68) when HbA_1c_ increased from 54 to 60 mmol/mol (7.1–7.6% NGSP), suggesting that uGMI may offer better risk discrimination in this range. As shown in Fig. 2, the steeper slope of the uGMI curve immediately after 54 mmol/mol (7.1% NGSP) further supports its stronger association with diabetic retinopathy risk compared with HbA_1c_.

### Decision tree analysis for diabetic retinopathy risk

**Fig. 3:**
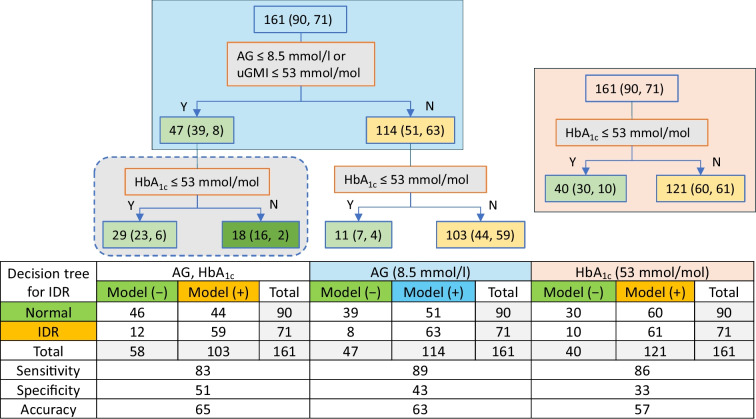
Decision tree models and performance metrics for predicting diabetic retinopathy. Nodes representing no diabetic retinopathy are coloured in green; nodes representing diabetic retinopathy are coloured in orange. Lighter shades indicate a smaller majority (i.e., the dominant class still exceeds 50%, but by a narrower margin). Blue and pink shading is used to indicate single variable decision trees with uGMI and HbA_1c_, respectively. In the decision tree boxes, the total number of participants in the node is shown, followed by the number of participants without diabetic neuropathy and number of participants with diabetic neuropathy. IDR, incident diabetic neuropathy

Decision tree models were constructed using the currently recommended glycaemic target of <53 mmol/mol (7.0% NGSP) for both HbA_1c_ and uGMI to evaluate their ability to classify diabetic retinopathy risk (Fig. 3). In individuals with higher uGMI values, adding an HbA_1c_ threshold of 53 mmol/mol (7.0% NGSP) increased specificity from 43% to 51%, but slightly reduced sensitivity from 89% to 83%. Among those with lower uGMI, individuals with HbA_1c_ values above 53 mmol/mol (7.0% NGSP) had a lower diabetic retinopathy rate compared to those with HbA_1c_ ≤53 mmol/mol (7.0% NGSP). Notably, the HbA_1c_ of most participants with diabetic retinopathy in the lower uGMI group was concentrated within a range of 48–58.5 mmol/mol (6.5–7.5% NGSP), suggesting a distinct glycaemic profile that may warrant further investigation.

### Logistic regression and random forest models for predicting diabetic retinopathy

To evaluate the independence of uGMI and HbA_1c_ in terms of their association with incident diabetic retinopathy, both linear models (logistic regression) and non-linear models (random forest) were used (ESM Fig. 2). In both models, the combination of uGMI and HbA_1c_ consistently achieved a modestly higher accuracy than individual parameters in classifying patients as having diabetic retinopathy. When used individually, uGMI and HbA_1c_ showed comparable performance. Non-linear models, such as random forests, outperformed the corresponding logistic regression models in all scenarios. The random forest models captured more complex relationships between glycaemic metrics and diabetic retinopathy risk, yielding higher accuracy. All observed differences in accuracy were statistically significant, with *p*<0.0001.

## Discussion

CGM metrics are essential for managing diabetes due to their ability to provide detailed information on glycaemic profiles and variations. Regulatory and clinical needs have driven the inclusion of CGM metrics in the ADA Standards of Care [10]. However, concern has been raised by the scientific and regulatory community regarding use of CGM metrics in trial outcomes due to a lack of compelling evidence like that available for HbA_1c_, which is a well-established biomarker of diabetes complications.

Recent studies have tried to evaluate the association of CGM metrics such as AG, TIR and TITR with diabetes complications using seven-point fingerstick data from the DCCT or by converting fingerstick data to virtual CGM-like data [2, 27]. These studies have shown a strong association of CGM metrics (especially AG, TIR and TITR) with microvascular complications. Using a unique approach, Shah et al [18] collected up to 7 years of retrospective continuous CGM data prior to development of diabetic retinopathy in adults with type 1 diabetes, and demonstrated similar diabetic retinopathy risk when assessed using HbA_1c_ as well as TIR and TITR.

In this analysis, we used robust statistical and machine learning models to evaluate the capability of CGM-derived metrics, especially uGMI, compared with HbA_1c_ for predicting diabetic retinopathy risk in adults with type 1 diabetes. We demonstrated that uGMI offers similar diabetic retinopathy risk prediction as HbA_1c_ does. uGMI outperformed other CGM-derived metrics such as TIR, TITR and others in distinguishing between individuals with and without diabetic retinopathy. Its strong predictive performance effectively overshadowed that of these other metrics, meaning that their contributions were minimal or redundant when uGMI was included in the model. Combined use of uGMI and HbA_1c_ led to a slight improvement in the classification and risk stratification of diabetic retinopathy. These small improvements, which correspond to approximately 2.5% or less in classification accuracy for both linear and non-linear models (Fig. 3 and ESM Fig. 2), are statistically significant but not clinically meaningful. This underscores the limited complementary role between HbA_1c_ and uGMI.

Our study offers useful clinical insights. While the current GMI formula, which is used to estimate HbA_1c_ from sensor glucose via linear regression, has been widely adopted, its limitations have been increasingly acknowledged. For example, GMI overestimates HbA_1c_ when mean glucose is below 7.2–7.8 mmol/l, and the relationship between GMI and HbA_1c_ within the same individual is not stable over time [28]. Studies have called for the GMI formula to be updated to provide better alignment with HbA_1c_ [13, 14]. Nevertheless, clinicians continue to value a metric that can approximate HbA_1c_, as such a metric facilitates discussions about glycaemic control with patients, particularly during virtual consultations. Our findings highlight a strong and consistent association between uGMI and diabetic retinopathy risk, closely mirroring that of HbA_1c_. Although a direct comparison between uGMI and GMI was not conducted in this study, the observed relationship with HbA_1c_ supports the potential value of uGMI. Based on this evidence, we suggest that uGMI may serve as a more informative alternative to GMI in CGM reports, such as the ambulatory glucose profile.

Despite robust methodology to evaluate the association of uGMI and HbA_1c_ with diabetic retinopathy, our research findings should be interpreted with caution. The limitations of this research include the relatively small sample size and the fact that it was a single-centre study and CGM data were collected retrospectively. Moreover, the cohort predominately comprised non-Hispanic white participants based on self-reported ethnicity, requiring cautious extrapolation of the results for other racial and ethnic groups. Despite these limitations, the study strengths include its longitudinal design, analysing CGM data collected over several years before the development of diabetic retinopathy, minimising biases that are inherent in cross-sectional studies. Additionally, the HbA_1c_ and CGM data used in this study span an extended period, providing a more accurate reflection of chronic glycaemic exposure and thus taking into account the associated time lag between glucose dysregulation and the onset of tissue damage.

In summary, CGM-derived uGMI is a strong predictor of diabetic retinopathy risk in adults with type 1 diabetes, and the risk prediction was similar to that for HbA_1c_. In addition, we found that uGMI and HbA_1c_ are not meaningfully complementary in differentiating participants who went on to develop diabetic retinopathy from those who did not.

## Supplementary Information

Below is the link to the electronic supplementary material.

ESM (PDF 300 KB)

## Data Availability

Data can be requested by contacting the corresponding author, indicating details of how the data are to be used. Data may be made available based on institutional agreements.

## Abbreviations

AG: Sensor mean glucose
CGM: Continuous glucose monitoring
GMI: Glucose management indicator
MIS: Mutual information score(s)
mRMR: Minimum redundancy maximum relevance
ROC: Receiver operating characteristics
TAR: Time above range
TBR: Time below range
TIR: Time in range
TITR: Time in tight range
uGMI: Updated glucose management indicator

## Authors’ relationships and activities

VNS’s institution has received research support from Dexcom, Eli Lilly, Enable Bioscience, Zucara Therapeutics, the Cystic Fibrosis Research Foundation, Breakthrough T1D and NIH. VNS reports receiving honoraria from Sanofi, NovoNordisk, Eli Lilly, Insulet, Tandem Diabetes Care, Dexcom, Ascensia Diabetes Care, Embecta, Biomea Fusion, Sequel Med Tech, Medtronic, Genomelink and Lumosfit for consultation, advice or speaking roles, outside the submitted work. All other authors are employees of Abbott Diabetes Care.
