# Supplementary material for: Association of HbA1c and an updated glucose management indicator (uGMI) with incident diabetic retinopathy in adults with type 1 diabetes: a longitudinal study
Source: Diabetologia. 2025 Nov 11;69(3):610–7. doi: 10.1007/s00125-025-06599-w (PMC12881093; doi:10.1007/s00125-025-06599-w)
Supplement: Supplementary file 1 — ESM (PDF 300 KB) [file 125_2025_6599_MOESM1_ESM.pdf]

ESM Methods

The relationship among AG, GMI and updated GMI

AG is a simple arithmetic average of all glucose readings over a period. With CGM, this usually means the average of glucose readings every 15 minutes.

Original GMI is calculated from AG as following:

$$GMI(mmol/mol) = 4.70587 * AG(mmol/l) + 12.71$$

or

$GMI(\%) = 0.02392 * AG(mg/dL) + 3.31$, which is a linear transformation of AG. Therefore, it should contain the same information as AG, i.e. having exactly same MIS and AUC ROC when correlating with another variable.

Updated GMI is calculated from AG through a nunlinear transformation:

$$updated\ GMI(mmol/mol) = [0.07808/AG(mmol/l)\ + 0.00389]^{-1} - 23.5$$

or

$updated\ GMI(\%) = (15.36/AG(mg/dL) + 0.0425)^{-1}$.

It contains very similar information as AG and maintains the same rank order. For this reason, it should produce similar MIS and ROC AUC as AG.

Tables

ESM Table 1. Characteristics of participants at time of study inclusion

	DR group (*N*= 71)	Control group (*N*= 92)
Age (years)		
Mean ± SD, range	27 ± 13 (14–76)	38 ± 16 (17–81)
Sex, n (%)		
Female	37 (52)	44 (48)
Male	34 (48)	48 (52)
Race/ethnicity, n (%)		
Non-Hispanic White	60 (85)	76 (83)
Other	9 (13)	5 (5)
Unknown	2 (3)	11 (12)
Health insurance, n (%)		
Private	56 (79)	79 (86)
Medicaid	14 (20)	10 (11)
Military plan	1 (1)	1 (1)
Unknown	0 (0)	2 (2)
BMI (kg/m^2)		
Mean ± SD	26 ± 4	25 ± 5
Underweight (<18.5 kg/m^2), n (%)	2 (3)	3 (3)
Normal weight (18.5 to <25.0 kg/m^2), n (%)	20 (28)	34 (37)
Overweight (25.0 to <30.0 kg/m^2), n (%)	27 (38)	29 (32)
Obese (≥30.0 kg/m^2), n (%)	11 (15)	11 (12)
Missing, n (%)	11 (15)	15 (16)
Duration of T1D (years)		
Mean ± SD, range	15 ± 6 (5–37)	20 ± 8 (4–47)
History of autoimmune disease, n (%)	13 (18)	22 (24)
History of cardiovascular disease, n (%)	1 (1)	0 (0)
Follow-up time (years)		
Mean ± SD, range	6.6 ± 1.5 (1.2–8.4)	6.5 ± 1.7 (1.1–9.1)

Figures

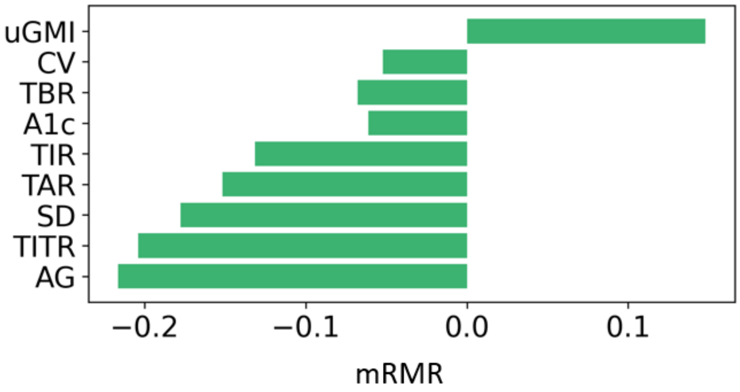

ESM Figure 1. Discriminative features between DR and control groups evaluated by minimum redundancy maximum relevance (mRMR) method

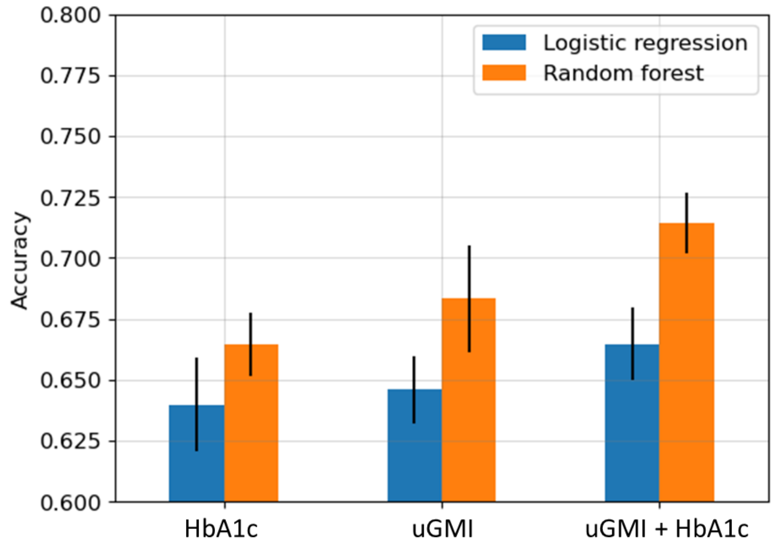

ESM Figure 2: Accuracy of models trained on HbA_{1c}, GMI, and combined metrics for predicting incident diabetic retinopathy
